# Flexural Behavior of Thin Concrete Slabs Reinforced with Surface Embossed Grid-Type Carbon-Fiber Composites

**DOI:** 10.3390/polym17030411

**Published:** 2025-02-04

**Authors:** Kyung-Min Kim, Sung-Woo Park, Kyung-Jae Min

**Affiliations:** 1 Seismic Safety Center, Korea Conformity Laboratories (KCL), 187, Saneop-ro 155 Beon-gil, Gwonseon-gu, Suwon-si 187, Gyeonggi-do, Republic of Korea; sungwoo@kcl.re.kr; 2 Korea Carbon Industry Promotion Agency, Jeonju 54853, Republic of Korea; bluemy@kcarbon.or.kr

**Keywords:** carbon grid, CFRP reinforcement, surface treatment, flexural behavior, pultruded CFRP strands, concrete compressive strength

## Abstract

Fiber-reinforced polymers (FRPs) are being increasingly used to replace rebars as reinforcements for concrete. In this study, the flexural behavior of one-way concrete slabs reinforced with a grid-type carbon FRP (CFRP) (carbon grid), in the form of strands with embossed surfaces, was experimentally investigated. The experimental variables included the effective depth, number of carbon grid layers, and concrete compressive strength. The results exhibit that the surface embossing of the CFRP strands effectively improves their bonding with concrete based on the crack formation pattern. Concrete specimens reinforced with carbon grids exhibited an increased maximum load and stiffness as the effective depth, number of carbon grid layers, and concrete compressive strength increased. Among the experimental variables, the effective depth exhibited the greatest influence on the flexural behavior of the carbon-grid-reinforced concrete specimen. Furthermore, the ratios of the experimental to calculated flexural strength values for all carbon-grid-reinforced concrete specimens ranged from 0.74 to 1.22. Based on the results, a trilinear load–deflection curve was proposed to simulate the flexural behavior of carbon-grid-reinforced concrete members, considering the bond property between the concrete and the carbon grid. The proposed trilinear load–deflection curve reasonably simulated the flexural behavior of the specimens reinforced with carbon grids.

## 1. Introduction

Fiber-reinforced polymers (FRPs) exhibit excellent chemical resistance, mechanical performance, structural properties, and strong corrosion resistance [1]. Therefore, to address the issue of chloride-induced damage [2,3,4] to reinforced concrete (RC) structures caused by steel corrosion, efforts to replace rebars with FRPs have been ongoing since 1970 [5,6]. FRP reinforcements have primarily been applied to bridge decks [5,6,7] exposed to marine environments that are particularly vulnerable to chloride damage. Recently, FRPs have been increasingly used in new constructions [8,9,10], including bridge foundations, building slabs, and walls, as well as for the repair and strengthening of existing concrete structures [11,12].

A FRP is a thermosetting or thermoplastic resin reinforced with fibers. The properties of FRPs depend on the type of resin and reinforcing fiber used. Glass FRPs (GFRPs) produced from thermosetting resins such as epoxy or vinyl ester reinforced with glass fibers are widely employed [5]. Recently, the use of carbon FRPs (CFRPs), which utilize carbon fibers because of their exceptional light weight, superior load-bearing strength at high temperatures, low density, high tensile strength and modulus, and excellent chemical resistance [13], has been increasing.

However, when cured by heating, thermosetting resins cannot be reshaped via reheating. This makes it difficult to bend FRP reinforcements made from thermosetting resins at construction sites, such as for stirrup hooks or anchoring the ends of longitudinal bars within a structure, as bending of FRP reinforcements must be carried out during the manufacturing process of the FRP reinforcement [14]. To facilitate bending at construction sites, some fiber-reinforced thermoplastic polymer (FRTP) reinforcements have been developed using thermoplastic resins including polyethylene, polypropylene, polyvinyl chloride, and polystyrene, which soften when heated and harden when cooled [15,16]. Most FRP/FRTP reinforcements have been developed with the same circular cross-section as their corresponding rebars.

On the other hand, textile-reinforced concrete (TRC) uses woven fibers as the reinforcement for concrete [17,18]. The flexural strength of TRC members has been reported to be proportional to the fiber orientation and amount of fiber in a particular direction [19,20]. Also, the tensile behavior of TRC members has been reported to be improved in terms of smaller crack gaps, enhanced stable crack geometry, and increased maximum load by impregnating or coating fibers with resin like FRP [21,22,23]. Recently, grid-type FRP reinforcements, in which continuous fibers are arranged in the 0° and 90° directions and impregnated or coated with resin, have been developed [8,9,10,21,22,23,24,25,26,27,28]. Thus, grid-type FRP reinforcements can be considered a type of TRC which comprise thin FRP strands with elliptical or flat cross-sections arranged at regular intervals in the warp and weft directions, with the intersections of the warp and weft strands integrated to form a grid shape [26,28]. Methods for integrating these intersections include knitting with additional fibers (biaxial warp-knitting structure) [26] or layering multiple FRP strands in the warp and weft directions and curing the resin (cross-laminate structure) [28]. Grid-type FRP reinforcements with a biaxial warp-knitting structure have been developed for both new construction and the repair or strengthening of existing structures. They are characterized by the relatively narrow spacing of the FRP strands, and high-strength mortar is primarily used instead of concrete [25,27]. Meanwhile, the grid-type FRP reinforcements with a cross-laminate structure have been developed for the repair or strengthening of existing structures. They are also used with mortar, despite having a maximum FRP-strand spacing of 100 mm.

Grid-type FRP reinforcements using thin FRP strands can effectively reduce the cross-sectional depths of structural members. Additionally, because the FRP strands are arranged in the warp and weft directions, they do not require the assembly of longitudinal and transverse reinforcements at construction sites, as is required for rebars or bar-type FRP reinforcements. Therefore, they are advantageous for use in new constructions and the repair or strengthening of existing structures. Accordingly, in addition to the evaluation of their bond with concrete through a pull-out test [29] for applying grid-type FRP reinforcements to concrete structures, research on the applicability of various types of concrete has been conducted for their application to different concrete members or structures. These include foamed concrete, which has excellent thermal insulation performance and low density due to the formation of numerous fine bubbles [30]; expansive concrete, which enhances crack behavior through chemical prestressing with expansive admixtures [31]; and engineered cementitious composites, which offer high-tensile properties using fibers [32].

Meanwhile, grid-type FRP reinforcements differ from bar-type FRP reinforcements regarding their cross-sectional shape, size, and spacing. Specifically, grid-type FRP reinforcements have smaller cross-sectional widths and thicknesses than bar-type FRP reinforcements, with a fixed spacing between the warp and weft FRP strands which is generally narrower than the spacing in steel-bar-type FRP reinforcements. Because untreated FRP surfaces are smooth [26], it is common practice to form ribs on the surface of bar-type FRP reinforcements, similar to steel bars, to improve their bonding with concrete [6]. In contrast, for grid-type FRP reinforcements, methods such as coating the surface with silica have been developed instead of forming ribs on the surface to improve bonding with concrete [33].

Unlike bar-type FRP reinforcements, grid-type FRP reinforcements differ not only in their material, but also in their cross-sectional and surface shapes, as well as their placement characteristics within concrete members. Consequently, the flexural behavior of concrete members reinforced with grid-type FRP reinforcements differs from that of concrete members reinforced with both bar-type FRP reinforcements and rebars [22,24]. Specifically, in a study of concrete members reinforced with grid-type FRP, when the tensile stress in the concrete tensile region reached the tensile strength, multiple cracks occurred continuously. After the initial crack, the load was temporarily decreased. It was then increased again, and the stiffness decreased significantly. The grid-type FRP reinforcement continued to resist the load until the flexural strength was reached, and the stiffness was significantly reduced compared to the initial stiffness before cracking.

After reaching the flexural strength, due to the brittle nature of FRPs, concrete members reinforced with grid-type FRP reinforcements exhibit brittle failure [8,22], similar to those reinforced with bar-type FRP reinforcements [34]. Accordingly, ACI 440.1R-15 [5] allows for flexural design, wherein concrete members reinforced with FRP reinforcements can attain their flexural strength through concrete crushing. To ensure the safety of FRP-reinforced flexural members against brittle failure due to FRP rupture or concrete crushing, strength reduction factors in the range of 0.55–0.65 have been suggested, depending on the failure mode. These strength reduction factors are primarily based on the experimental results of concrete members reinforced with steel-bar-type GFRPs. However, flexural tests on concrete members reinforced with grid-type CFRP reinforcements have shown a lower flexural performance than the abovementioned strength reduction factors [35]. Although the flexural performance of concrete members reinforced with FRPs can vary depending on the type of reinforcing fibers and FRP reinforcements, experimental studies on this topic are limited [28,35].

Therefore, this study aimed to evaluate the performance of a grid-type CFRP reinforcement (carbon grid), KC, developed using thin, flat CFRP strands made from carbon fiber and epoxy through pultrusion. These strands were embossed to increase their bonding with concrete, arranged in the warp and weft directions, and bonded together with an adhesive to form grid-type CFRP reinforcements [35]. One-way concrete slab specimens reinforced with carbon grid KC were fabricated, and experimental variables, including the effective depth, corresponding amount of carbon grid reinforcement, number of carbon grid layers, and concrete compressive strength, were evaluated. The flexural behavior of members reinforced with carbon grid KC was evaluated using a three-point bending test. A three-point bending test was conducted on one-way RC slab specimens to evaluate and compare the performance of carbon grid KC as a reinforcement for concrete with that of a rebar.

## 2. Materials and Methods

### 2.1. Experimental Design

#### 2.1.1. Material Properties

**Figure 1:**
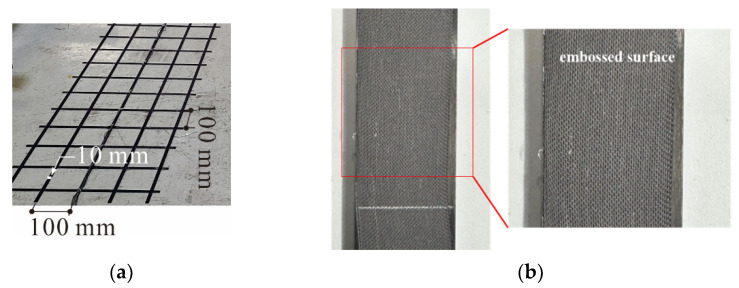
Images of carbon grid: (**a**) overall appearance; (**b**) surface of carbon-fiber-reinforced polymer (CFRP) strand.

Figure 1 presents the carbon grid, KC, used in this study. Carbon grid KC was manufactured by first producing the CFRP strands through pultrusion and then bonding them together with an adhesive using automated production equipment.

The carbon fiber volume fraction of the CFRP strand was designed to be 70 vol.%. High-tensile-strength carbon fiber (Hyosung, H2550 12 K) and a three-component low-viscosity liquid epoxy-based system (Huntsman, ARALDITE^®^ CY 5192-1) were used as the reinforcing fiber and resin, respectively. The resin was Bisphenol A-Epoxy Cyclohexane-Methyl Carbonate-based Epoxy designed for pultrusion and high-temperature (the glass transition temperature, T_g_ > 210 °C) application. As a hardener, Methyl-5-norbornene-2,3-dicarboxylic anhydride, which has good high-temperature resistance, was used to enhance heat resistance. Therefore, as an accelerator, methylimidazole, which is commonly used as an accelerator for acid anhydride-based epoxy resins, was used. The dynamic mechanical analysis (DMA) results exhibited that the T_g_ was 226.5 °C. The resin was used in a ratio of 100:131:6 with the base, hardener, and accelerator.

Carbon fibers were impregnated with epoxy-based resin. In the mold, the epoxy-based resin was then cured under conditions of 155 °C at the inlet, 205 °C at the center, and 205 °C at the outlet, and produced strands with a width of 10 mm and thickness of 1 mm through pultrusion. To enhance their bonding with concrete, the surfaces of the CFRP strands were embossed to increase their specific surface area (Figure 1b). Peel ply surface treatment, which imparts a textured surface formed by alternating the weft and warp threads of the CFRP strands, was applied [36]. Specifically, peel ply (polyester taffeta woven fabric, with a weight of 85 g/m^2^ and a maximum heat resistance temperature of 210 °C) was laid upon the outer surfaces of the CFRP strands during pultrusion and peeled off before forming the grid with the CFRP strands. The peel ply absorbed some of the epoxy-based resin and became an integral part of the CFRP strands during pultrusion. Then, by peeling the peel ply off, the epoxy-based resin was fractured, and an embossing was formed on the surfaces of the CFRP strands.

To form the grid with the CFRP strands, Methyl-Methacrylate (MMA) adhesive (Bostik, SAF 30-15), a structural adhesive, was used. The MMA adhesive was characterized by a fast fixture time of 11 to 17 min at 24 °C and a high lap shear strength of over 20 MPa. The carbon grid was manufactured by bonding the horizontal and vertical CFRP strands with the MMA adhesive. Specifically, the CFRP strands were first arranged horizontally at 100 mm intervals. After applying the MMA adhesive at the intersection points of the horizontal and vertical CFRP strands, the vertical CFRP strands were also arranged at 100 mm intervals. Finally, the intersection points were compressed to form a grid [37].

**Figure 2:**
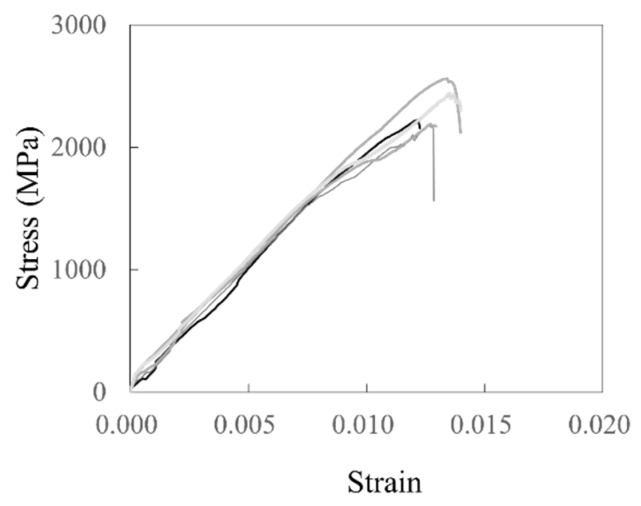
Material test results for CFRP strand samples.

Table 1 summarizes the characteristics of carbon grid KC and the rebar, as well as the tensile test results of the CFRP strands with carbon grid KC and the rebar. The tensile strengths and moduli of the CFRP strands were determined considering ASTM D7205 [38] and CAN/CSA-S806-02 [39] as references. The tensile specimens were prepared for these tests by attaching steel plates to both ends of the CFRP strands and filling the spaces between the plates with epoxy. A universal testing machine (UTM) with a capacity of 1000 kN was used to apply the load at a speed of 5 mm/min. Tensile tests were conducted on the rebar according to ISO 6935 [40], and the yield strength, tensile strength, and tensile modulus were determined. The tensile strength of the CFRP strands in carbon grid KC was found to be 420.8% higher than that of the rebar, while the tensile modulus was similar to that of the rebar. In addition, the tensile stresses in the CFRP strands sharply decreased after reaching the tensile strength (Figure 2).

**Figure 3:**
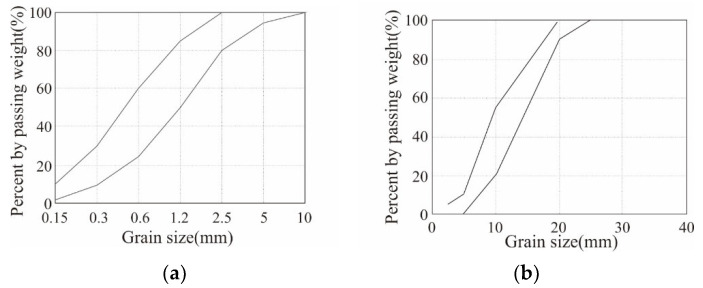
Standard particle-size distribution curves: (**a**) fine aggregate; (**b**) coarse aggregate.

**Figure 4:**
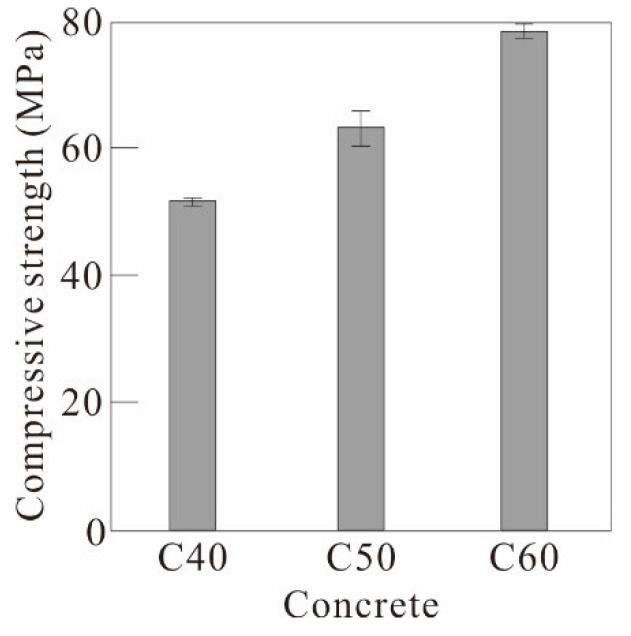
Material test results for concrete samples.

Table 2 summarizes the mix design and 28-day compressive strength of the concrete. Three types of concrete were used: C40, C50, and C60. To achieve target compressive strengths of 40, 50, and 60 MPa, the water–cement ratio of the concrete was adjusted from 38% to 25%. Coarse and fine aggregates were used according to the standard particle-size distribution specified in KS F 2527 [41] (Figure 3), with a maximum aggregate size of 25 mm for the coarse aggregate. From the results of compression tests conducted in accordance with ISO 1920-4 [42], the average 28-day compressive strengths of the C40, C50, and C60 concretes were 51.7, 63.3, and 78.7 MPa, respectively, exceeding the designed compressive strengths (Table 2 and Figure 4).

#### 2.1.2. Specimen Overview

**Figure 5:**
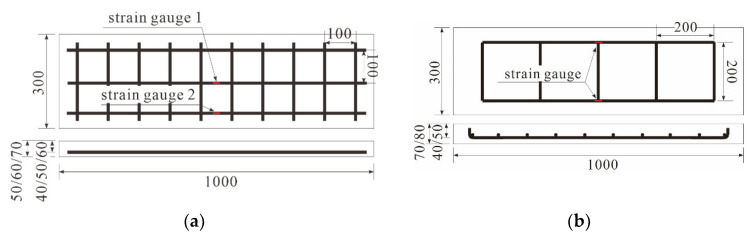
Diagrams of specimens reinforced with (**a**) carbon grids, (**b**) rebars.

To evaluate the applicability of carbon grid KC in concrete structures, one-way slab specimens reinforced with carbon grid KC were fabricated (Table 3 and Figure 5). The primary experimental variables included the effective depth of the specimens, corresponding amount of carbon grid reinforcement, number of carbon grid layers, and concrete compressive strength. One-way RC slab specimens with varying concrete compressive strengths were prepared for the comparison of carbon grid KC with rebars, which are commonly used as reinforcements for concrete. Carbon grids are chemically stable materials [43] with excellent corrosion resistance to chloride-ion penetration. Unlike in RC structures, there is no need to ensure a minimum cover thickness to prevent chloride-induced damage. Therefore, the cover thickness of the carbon-grid-reinforced specimens was set to 10 mm.

**Figure 6:**
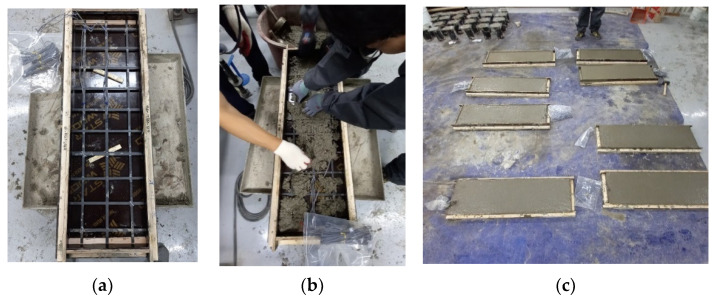
Manufacturing process for specimens reinforced with carbon grids: (**a**) arrangement of carbon grid; (**b**) concrete pouring; (**c**) completion of concrete pouring.

To fabricate the carbon-grid-reinforced specimens, a formwork was initially placed on top of a vibration compactor, as shown in Figure 6. Wooden plates (10 mm thick) were installed at both ends of the formwork to secure the cover thickness. The carbon grid was placed on wooden plates, concrete was poured over the carbon grid, and a vibration compactor was used to ensure that the concrete filled the space below the carbon grid without any voids. The formwork was removed 1 day after concrete placement, and the specimens were cured indoors for at least 28 days. When installing carbon grids at construction sites, devices such as spacers must be installed to secure the carbon grids in place [44]. However, in this study, due to the small specimen size, wood plates were used instead of spacers to fix the carbon grid at the designed cross-sectional positions, considering the specimen size and the potential influence of spacers.

Table 4 summarizes the crack and flexural strengths of the specimens, calculated based on the material test results in Table 1 and Table 2, assuming the equilibrium of forces and strain compatibility [5]. Here, the crack strength and flexural strength refer to the strengths at which the crack initiated and the load reached its maximum, respectively.

Among the carbon-grid-reinforced specimens, specimens 40_KC_d50 and 60_KC2_d40 were over-reinforced with carbon grids, with reinforcement-to-balanced reinforcement ratios ($\rho /\rho_{b}$) of 1.35 and 1.87, respectively. Therefore, compression failure due to concrete crushing at the compression edge was expected to occur before the carbon grid ruptured. Specimen 40_KC_d60 was under-reinforced with carbon grids, with a reinforcement-to-balanced reinforcement ratio ($\rho /\rho_{b}$) of 0.69, and failure due to carbon grid rupture was expected. Meanwhile, for specimens 50_KC_d40 and 60_KC_d40, the reinforcement-to-balanced reinforcement ratios ($\rho /\rho_{b}$) were 0.96 and 0.94, respectively, which are only slightly lower than their balanced reinforcement ratios. Therefore, failure due to the rupture of the carbon grid was expected.

For RC specimens 40_RC_d50, 50_RC_d40, and 60_RC_d40, the rebars were arranged to achieve a flexural strength similar to that of the carbon-grid-reinforced specimens with the same effective depth and concrete compressive strength. Consequently, final failure due to steel yielding was expected for all RC specimens.

### 2.2. Loading and Measurement Method

**Figure 7:**
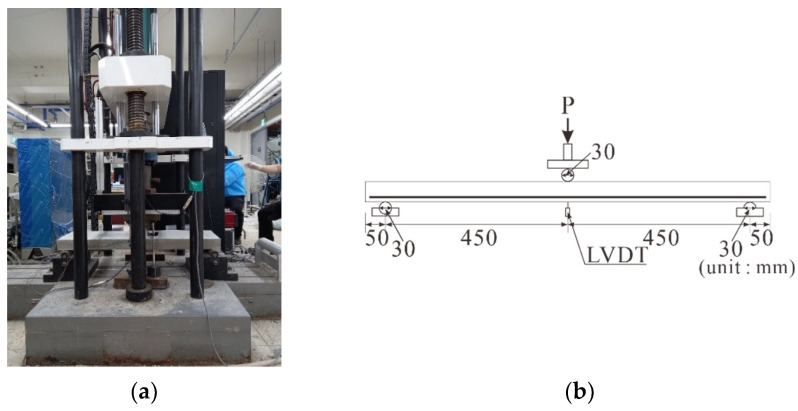
Test setup: (**a**) picture of universal testing machine; (**b**) schematic of loading and measurements.

The specimens were loaded using a 1000 kN UTM at the center of the specimen in a three-point bending setup with a displacement control rate of 5 mm/min, as presented in Figure 7a.

The load applied on the specimens was measured using a load cell integrated into the UTM, and the deflection was measured using two linear variable displacement transducers (LVDTs) (Tokyo Measuring Instruments Lab, Tokyo, Japan) installed at both ends of the midsection at the bottom of the specimen, as illustrated in Figure 6b. The strain in the CFRP strands of the carbon grid was measured using strain gauges [12] attached to the surfaces of both the CFRP strands and rebars to determine their deformation. Specifically, for the carbon grid, strain gauges were attached to the top and bottom surfaces at two locations: the midpoints of two CFRP strands located at the end and center of the specimen (Figure 5a). Similarly, for the rebars, strain gauges were attached to the top and bottom surfaces of the two rebars at the center of the specimens (Figure 5b).

The concrete damage on the surfaces of the specimens, such as cracks, crushing, and spalling, was visually observed.

## 3. Results and Discussion

### 3.1. Crack and Failure Geometry

**Figure 8:**
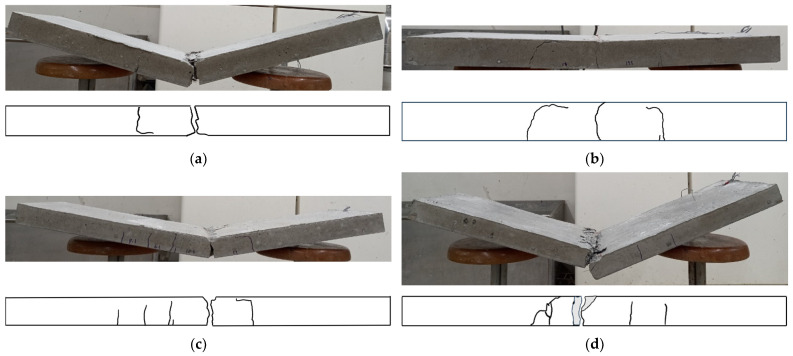

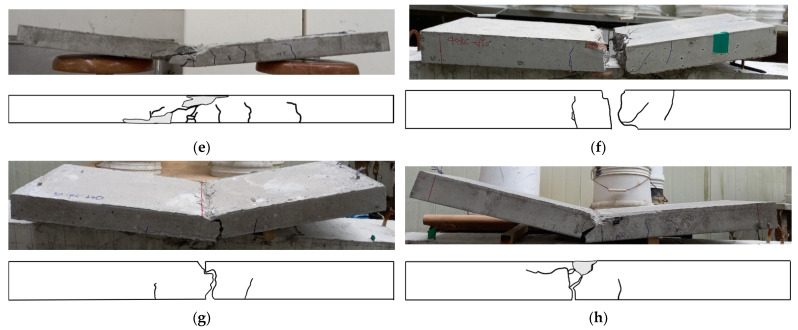
Failure of specimens: (**a**) 40_KC_d50; (**b**) 40_KC_d60; (**c**) 50_KC_d40; (**d**) 60_KC_d40; (**e**) 60_KC2_d40; (**f**) 40_RC_d50; (**g**) 50_RC_d40; (**h**) 60_RC_d40.

Table 5 summarizes the number of cracks observed on the surfaces of the specimens, while Figure 8 illustrates the surface damage and final failure modes after testing.

As a result of the load application, the first bending crack on the surfaces of the carbon-grid-reinforced specimens occurred just below the loading point, where the bending moment was greatest. As the load increased, the existing crack widened, and the load was transferred through the bonding stress between the CFRP strands and the concrete [45]. Subsequently, new bending and bending-shear cracks formed in the direction of the supports, where the bending moment decreased, and the influence of the shear force became more significant, once the stress at the tensile edge of the section reached the tensile strength of the concrete. Concrete crushing at the compression edge of the section occurred with continuous loading, and the carbon-grid-reinforced specimens ultimately failed due to the rupture of the longitudinal CFRP strands or the spalling of the cover concrete.

The number of cracks decreased in the RC specimens as the concrete compressive strength increased, with three, three, and two cracks forming at compressive strengths of 51.7 (C40), 63.3 (C50), and 78.7 MPa (C60), respectively. In contrast, the number of cracks increased in the carbon-grid-reinforced specimens, with 2–3 cracks for C40, 5 cracks for C50, and 5–6 cracks for C60 concrete, as the compressive strength increased. Consequently, except for the specimens comprising C40, the carbon-grid-reinforced specimens exhibited 1.67–3.00 times more surface cracks than the RC specimens. Unlike in previous experiments [35,36], no bond cracks formed at the carbon grid location in the carbon-grid-reinforced specimens. Instead, the flexural and flexural-shear cracks spread from the center to the ends of the specimens, indicating that the surface embossing treatment of the carbon grid (Figure 1b) effectively improved the bonding between the concrete and the carbon grid when considering only the surface damage occurrence.

Further, the carbon-grid-reinforced specimens exhibited increased surface damage, such as spalling of the cover concrete, as the concrete compressive strength increased. Additionally, the under-reinforced specimens, 40_KC_d60, 50_KC_d40, and 60_KC_d40, failed due to the rupture of the longitudinal CFRP strands. Meanwhile, among the over-reinforced specimens, 40_KC_d50 exhibited a longitudinal CFRP strand rupture as the final failure mode. Due to the concrete damage such as cracking and concrete spalling as well as the rupture of the longitudinal CFRP strands in the carbon grid, plastic deformation occurred after the tests.

**Figure 9:**
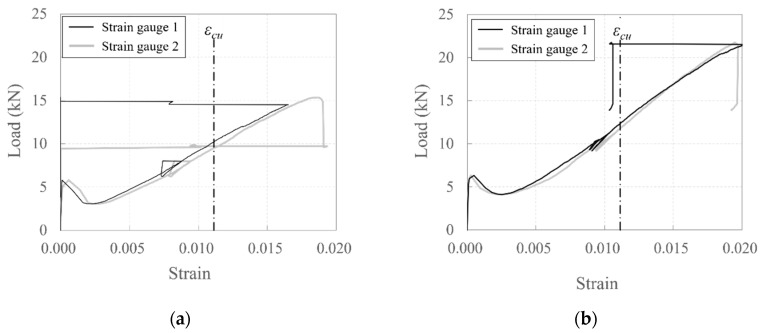

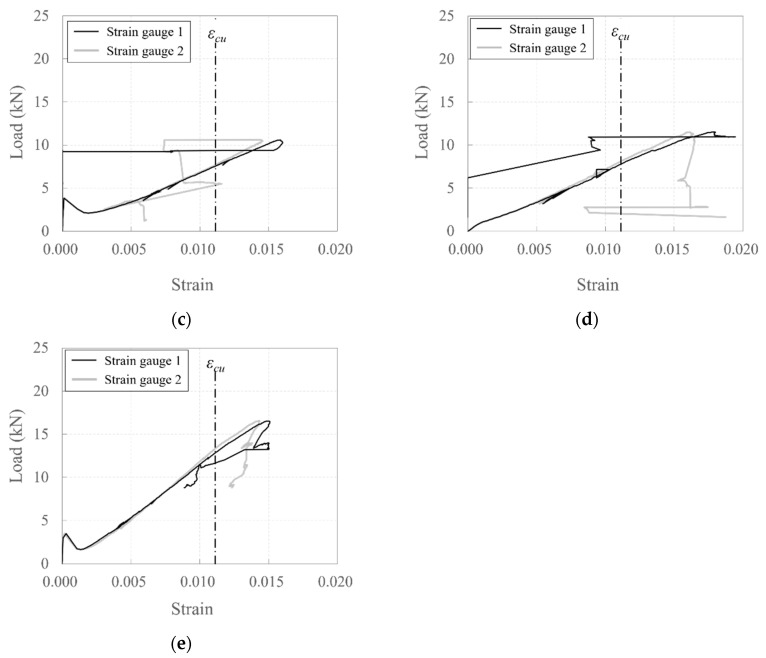
Plots of relationship between load and strain for specimens: (**a**) 40_KC_d50; (**b**) 40_KC_d60; (**c**) 50_KC_d40; (**d**) 60_KC_d40; (**e**) 60_KC2_d40.

Figure 9 depicts the relationship between strain and load for the carbon-grid-reinforced specimens. The strain was measured using four strain gauges attached to the surfaces of the longitudinal CFRP strands (Figure 5a), and $\varepsilon_{cu}$ represents the tensile strain at the tensile strength, which is obtained by dividing the tensile strength by the tensile modulus of elasticity. The black and gray curves, respectively, represent the average strain values measured by two strain gauges attached to the surface of the longitudinal CFRP strand at the center and end positions of the cross-section (Figure 5a).

In all carbon-grid-reinforced specimens, strain values exceeding the tensile strain ($\varepsilon_{cu}$) at the tensile strength were observed at 55–81% of the maximum load. This indicates that, in all specimens, the longitudinal CFRP strands reached their tensile strength before the specimens reached the maximum load, specifically at load levels ranging from 55% to 81% of the maximum load. According to previous experimental results [35], strain exceeding the tensile strain at the tensile strength could occur because of interfacial failure between the fibers and resin, suggesting that damage such as interfacial failure between the fibers and resin in the CFRP strands occurred before the crushing of the concrete at the compression edge in the specimens. For all specimens, no differences in strain were observed based on the locations of the strain gauge attachments. On the other hand, the strains of specimens 50_KC_d40 and 60_KC2_d40, where the damage occurred was relatively distributed, were smaller than those of other specimens.

### 3.2. Load–Deflection Relationship

Table 6 and Table 7 summarize the load, mid-span deflection, and stiffness of the specimens at each stage. The flexural behavior of the carbon-grid-reinforced members can be divided into three stages: pre-crack, crack formation (where cracks form continuously), and crack stabilization (where the flexural strength is reached without further crack formation) [22]. The crack stabilization points in Table 6 refer to the last crack formation point, which marks the beginning of the crack stabilization stage. The stiffness at each stage in Table 7 refers to the slope of the line connecting the origin to the crack initiation point, the crack initiation point to the crack stabilization point, and the crack stabilization point to the maximum load point.

For specimens 40_KC_d50 and 40_KC_d60, which had the same concrete compressive strength but different effective depths, the loads at the crack point, crack stabilization point, and maximum load point increased by 1.09, 1.41, and 1.41 times, respectively, as the effective depth increased. However, the ratio of the crack stabilization point load to the maximum load remained the same. Generally, the flexural stiffness of a member increases in proportion to the moment of inertia as the section depth increases [46,47]. After cracking, the stiffnesses of specimen 40_KC_d60, which had a larger effective depth, were 1.51 and 2.10 times greater than those of specimen 40_KC_d50.

With the same effective depth but different concrete compressive strengths, the load ratios for specimens 50_KC_d40 and 60_KC_d40 compared to that for specimen 30_KC_d40 are 1.20 and 2.61 at the crack point, 1.91 and 1.67 at the crack stabilization point, and 1.37 and 1.50 at the maximum load point, exhibiting an overall increase in load at these points with increasing concrete compressive strength. Among these, the highest stiffness was observed in specimen 50_KC_d40, which comprised concrete with a compressive strength of 63.3 MPa (C50), followed by specimen 60_KC_d40, which contained concrete with a compressive strength of 78.7 MPa (C60). This suggests that the stiffness increases with the concrete compressive strength before decreasing beyond a certain point.

For specimens reinforced with a single layer of the carbon grid, the maximum load increased with larger effective depths, likely because all the specimens failed due to the rupture of the longitudinal CFRP strands, and with a larger effective depth, the distance from the neutral axis to the carbon grid increased.

With the same concrete compressive strength and effective depth but different numbers of carbon grid layers, the load at the crack point was higher in specimen 60_KC_d40, comprising one carbon grid layer, compared with 60_KC2_d40. However, the loads in the crack stabilization and maximum load points were higher in specimen 60_KC2_d40, which had two layers of carbon grids. This is attributed to the increased tensile strength resulting from the doubling of the cross-sectional area of the longitudinal CFRP strands in 60_KC2_d40, thereby increasing the maximum load. Additionally, cracks propagated from the center to the ends under higher loads after the crack formation load due to the increase in the maximum load.

**Figure 10:**
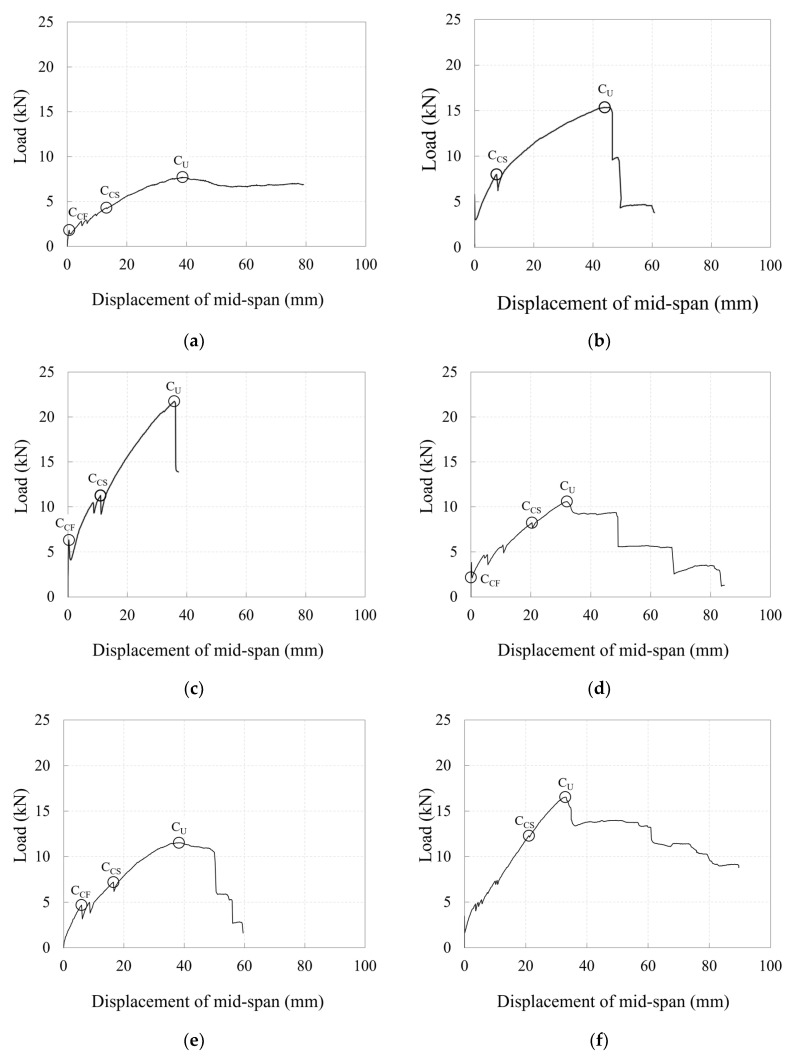
Plots of relationship between load and mid-span deflection for specimens: (**a**) 30_KC_d40; (**b**) 40_KC_d50; (**c**) 40_KC_d60; (**d**) 50_KC_d40; (**e**) 60_KC_d40; (**f**) 60_KC2_d40.

Figure 10 depicts the load–mid-span deflection relationships for the specimens.

Specimens 40_KC_d50 and 40_KC_d60, which had greater depths, were expected to have higher crack strengths (Table 4), and the experimental results exhibited that the loads at their crack formation points were also higher compared to the specimens with smaller depths (Table 6). Indeed, specimens 40_KC_d50 and 40_KC_d60 exhibited fewer cracks and larger crack spacings compared to the specimens with smaller depths (Figure 8). Consequently, the load concentrated on the bending crack at the loading point, causing the crack width to increase. As a result, a greater tensile force was generated in the longitudinal CFRP strands, attempting to pull them out. When the maximum load was reached simultaneously with concrete crushing at the compression edge, the longitudinal CFRP strands ruptured, leading to a sharp decrease in the load. In fact, larger strains (stresses) were observed when the maximum load was reached compared to the specimens with smaller depths (Figure 9).

Specimens 50_KC_d40, 60_KC_d60, and 60_KC2_d60, which had smaller depths, exhibited more cracks and smaller crack spacings compared to the specimens with greater depths (Figure 8), indicating that the load was distributed across multiple cracks. Consequently, smaller strains (stresses) were observed when the maximum load was reached (Figure 9). Meanwhile, for specimen 60_KC_d60, the cover concrete spalled at the loading point, and when the maximum load was reached, significant strains (stresses) were observed on the longitudinal CFRP strands, causing a sharp decrease in the load after the maximum load was reached compared to the other specimens. Additionally, specimen 60_KC2_d40, reinforced with two layers of carbon grids, exhibited a gradual decrease in load after reaching its maximum load due to the spalling of the cover concrete.

Under-reinforced specimens 40_KC_d60, 50_KC_d40, and 60_KC_d40 exhibited brittle failure, with the longitudinal CFRP strands rupturing after reaching the maximum load, causing the load to decrease sharply. In specimen 40_KC_d60, the load decreased sharply as soon as the maximum load was reached due to the rupture of the longitudinal CFRP strands. In contrast, for specimens 50_KC_d40 and 60_KC_d40, after reaching the maximum load due to concrete crushing the load was maintained for a short period as it was transferred to the carbon grid. However, it decreased sharply each time a longitudinal CFRP strand ruptured. Specimen 50_KC_d40 exhibited a larger deformation range in which the longitudinal strands maintained the load, compared with specimen 60_KC_d40. This was likely due to less concrete spalling in specimen 50_KC_d40 (Figure 8) and its lower maximum load.

Among the specimens over-reinforced with carbon grids, specimens 30_KC_d40 and 60_KC2_d40 experienced load drops of 86% and 81% of their maximum loads, respectively, after reaching their maximum loads due to concrete crushing. However, the load gradually decreased as it was transferred to the carbon grid until the end of the test. Specimen 40_KC_d50, which was over-reinforced, with a reinforcement-to-balanced reinforcement ratio of 1.35 (Table 4), was expected to fail due to concrete crushing. However, the test results exhibited that the longitudinal CFRP strands ruptured at the maximum load, resulting in brittle failure with a sharp decrease in the load. Specimen 40_KC_d50 exhibited fewer cracks than the other specimens (Figure 8a), with the damage concentrated in the central region. Notably, the cracks at the center of the specimen widened across the entire section, causing spalling of the cover concrete at the bottom of the carbon grid in the tensile region, which concentrated the tensile force on the longitudinal CFRP strands, leading to their rupture.

**Figure 11:**
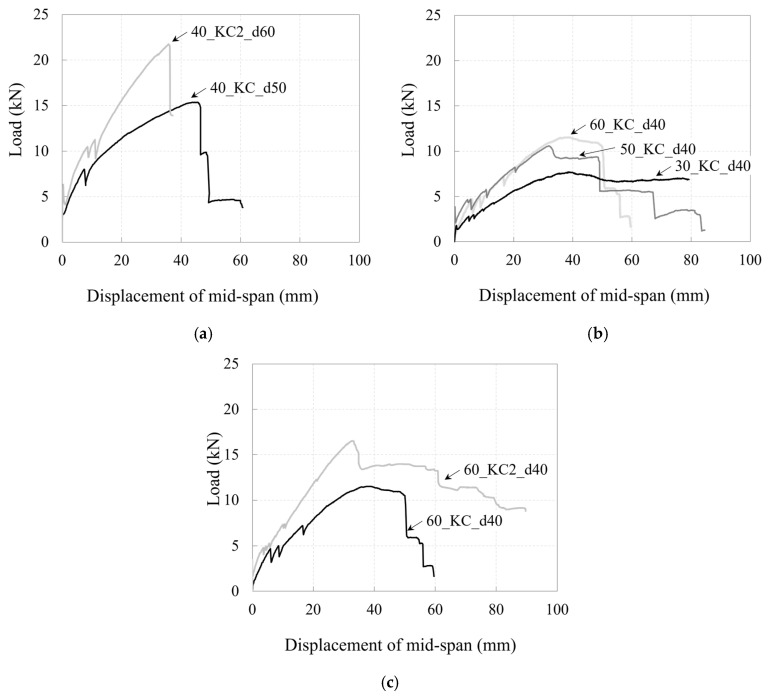
Plots of relationship between load and mid-span deflection for specimens considering (**a**) effective section depth; (**b**) concrete compressive strength; and (**c**) carbon grid layers.

Figure 11 demonstrates a comparison of the load–mid-span deflection relationships for the specimens based on the effective depth, concrete compressive strength, and number of carbon grid layers.

As the effective depth increased, the moment of inertia of the section also increased, leading to greater stiffness in the specimens, particularly after crack initiation. As a result, the specimens reached the maximum load at smaller deflections and achieved a higher maximum load.

Specimen 30_KC_d40, which had the lowest concrete compressive strength, exhibited lower post-cracking stiffness than the other specimens; because the longitudinal CFRP strands did not rupture [36], the load gradually decreased in a ductile manner after reaching the maximum load until the end of the test. In contrast, specimens 50_KC_d40 and 60_KC_d40, which had relatively higher concrete compressive strengths, exhibited similar stiffnesses from crack initiation to the maximum load, although specimen 60_KC_d40 had a higher maximum load. After reaching the maximum value, the load decreased as the longitudinal CFRP strands ruptured.

When more carbon grid layers were incorporated, the cross-sectional area of the carbon grid increased, leading to higher stiffness in the specimens. These specimens reached their maximum load at smaller deflections and exhibited higher maximum loads. Specimen 60_KC_d40, with one carbon grid layer, exhibited brittle behavior after the rupture of the longitudinal CFRP strands at the maximum load, whereas specimen 60_KC2_d40, with two carbon grid layers, experienced a gradual load decrease after the maximum load because of damage, i.e., concrete spalling.

### 3.3. Crack and Maximum Load

**Figure 12:**
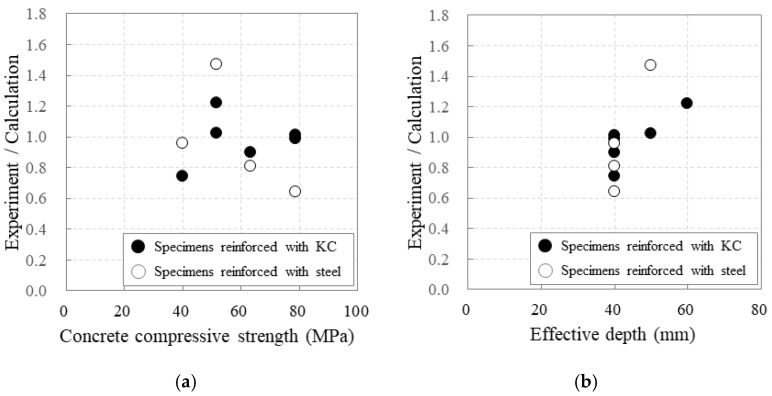
Flexural strength ratios of experimental and calculation results according to (**a**) concrete compressive strength and (**b**) effective depth.

The ratio of the experimental to calculated maximum load increased as the effective depth of the specimens increased (Figure 12). For specimens 40_KC_d50 and 40_KC_d60, the experimental maximum loads were 102% and 122% of the calculated maximum loads, respectively; thus, the experimental values were similar to or greater than the calculated values. Unlike the RC specimens, for the carbon-grid-reinforced specimens, the ratios of the experimental to calculated maximum loads increased as the concrete compressive strength increased. For specimens 60_KC_d40 and 60_KC2_d40, the experimental maximum loads were 99% and 101% of the calculated maximum loads, respectively, indicating similar experimental and calculated values. Therefore, specimens 40_KC_d60, 60_KC_d40, and 60_KC2_d40 demonstrated the same failure patterns (Figure 8 and Figure 10) and maximum loads, as summarized in Table 4, indicating that the design performance targets were achieved.

**Figure 13:**
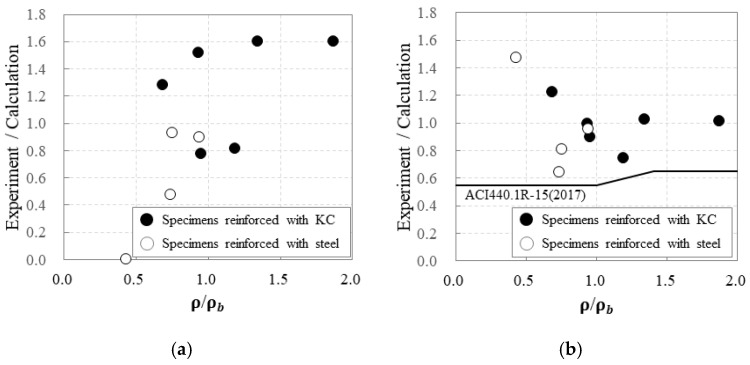
Strength ratios of experimental and calculation results according to $\rho_{g}/\rho_{gb}$: (**a**) crack; (**b**) flexural strengths.

Figure 13 demonstrates a comparison of the ratios of the experimental to calculated values for the crack and maximum loads based on the reinforcement-to-balanced reinforcement ratio.

For the RC specimens, the ratios of the experimental to calculated crack loads ranged from 0.47 to 0.93. As the reinforcement-to-balanced reinforcement ratio increased, the ratio of the experimental to calculated crack loads also tended to increase, though the experimental crack load was generally lower than the calculated result. In contrast, for the carbon-grid-reinforced specimens, the ratios of the experimental to the calculated crack loads ranged from 0.77 to 1.60. As the reinforcement-to-balanced reinforcement ratio increased, the ratio of the experimental to calculated crack loads tended to increase, and, except for specimens 30_KC_d40 and 50_KC_d40, the experimental crack loads were greater than the calculated results.

Regarding the RC specimens, the maximum load did not exhibit a clear trend with the reinforcement-to-balanced reinforcement ratio, and, except for specimen 30_RC_d40 which experienced rebar rupture, the ratios of the experimental to calculated maximum loads were low, ranging from 0.64 to 0.96. Notably, for 60_RC_d40, which had the highest concrete compressive strength, the ratio of the experimental to the calculated maximum load was the lowest.

In contrast, for the carbon-grid-reinforced specimens, for those under-reinforced with carbon grids, the ratios of the experimental to calculated maximum loads decreased as the reinforcement-to-balanced reinforcement ratio increased. For those over-reinforced with carbon grids, the ratios of the experimental to calculated maximum loads increased as the reinforcement-to-balanced reinforcement ratio increased. Meanwhile, ACI 440.1R-15 [5] recommends applying strength reduction factors of 0.55–0.65 for flexural members reinforced with FRP reinforcements, considering the failure modes based on the reinforcement condition of the FRP within the section to ensure safety. From this experiment, the ratios of the experimental to calculated maximum loads for the carbon-grid-reinforced specimens range from 0.74 to 1.22 (Figure 12b), which were 1.24–2.22 times the strength reduction factor recommended by ACI 440.1R-15 [5].

### 3.4. Flexural Behavior Evaluation

#### 3.4.1. Flexural Behavior Model

**Figure 14:**
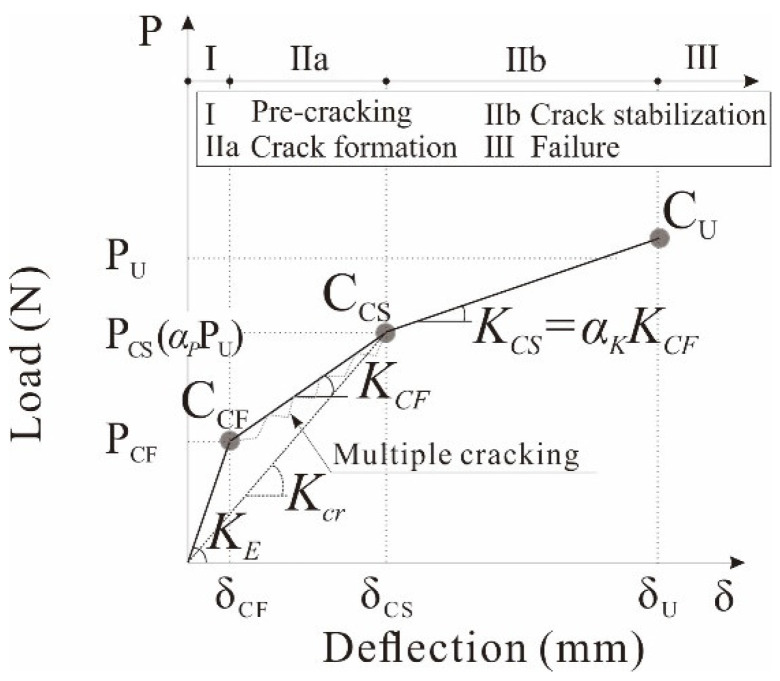
Trilinear load–deflection curve.

The flexural behavior of the carbon-grid-reinforced members can be divided into three stages: pre-crack, crack formation, and crack stabilization [22]. As summarized in Table 7, the stiffness of the carbon-grid-reinforced specimens decreased due to crack initiation, and further decreases in stiffness were observed during the crack stabilization stage. Accordingly, the flexural behavior of the carbon-grid-reinforced specimens can be assumed to be a trilinear curve that has three characteristic points—the crack formation point, *C_CF_*; crack stabilization point, *C_CS_*; and maximum load point, *C_U_*—and whose stiffness changes around the characteristic points of the crack formation point and crack stabilization point, as illustrated in Figure 14.

Here, the crack formation load, *P_CF_*, maximum load, *P_U_*, initial stiffness before crack formation, *K_E_*, and crack formation deflection, *δ_CF_*, can be evaluated similarly to those of the RC members. The stiffness in the crack formation stage, *K_CF_*, can also be evaluated as the slope of the line connecting the crack initiation point to the crack stabilization point using Equations (1) and (2) as follows:

(1) $$K_{CF}=\frac{P_{CS}-P_{CF}}{\left(\frac{1}{K_{cr}}\right)P_{CS}-\delta_{CF}}$$

(2) $$K_{cr}=\frac{48E_{c}I_{e}}{L^{3}}$$

where *P_CS_* is the load at the crack stabilization point (N), *P_CF_* is the load at the crack formation point (N), *δ_CF_* is the deflection at the crack formation point (mm), *Kcr* is the post-cracking stiffness (N/mm), *E_c_* is the modulus of elasticity of the concrete (MPa), *I_e_* is the effective moment of inertia (mm^4^), and *L* is the specimen span length (mm).

According to previous experimental results [35], one-way concrete slabs reinforced with carbon grid reinforcements exhibited lower post-cracking stiffness than RC members and even bar-type FRP reinforcements due to the weak bond between the carbon grid reinforcements and the concrete, making it difficult to expect the tension-stiffening effect of concrete. Therefore, the following Equations (3) and (4) for the effective moment of inertia were proposed to evaluate the stiffness reduction in the carbon-grid-reinforced members after cracking [35]. The stiffness reduction factor $\beta_{d}$ of 0.05 was proposed based on previous experimental results.

(3) $$I_{e}={\left(\frac{M_{cr}}{M_{a}}\right)}^{3}\beta_{d}I_{g}+(1-{\left(\frac{M_{cr}}{M_{a}}\right)}^{3})I_{cr}$$

(4) $$\beta_{d}=0.01\frac{\rho_{f}}{\rho_{fb}}\leq I_{g}$$

where *M_cr_* is the cracking moment (Nmm), *M_a_* is the applied service load moment (Nmm), $\beta_{d}$ is the stiffness reduction factor, *I_g_* is the gross moment of inertia (mm^4^), *I_cr_* is the cracked transformed moment of inertia (mm^4^), $\rho_{f}$ is the FRP reinforcement ratio, and $\rho_{fb}$ is the FRP reinforcement ratio producing balanced strain conditions.

As summarized in Table 8, the calculated stiffnesses in the crack formation stage ranged 0.89~1.44 of the experimental stiffness in the crack formation stage. Particularly, the ratios of the experimental to calculated stiffness in the crack formation stage tended to be higher for the over-reinforced specimens. As a result, the trilinear load–deflection curve in Figure 13 can be predicted if the characteristic values of the crack stabilization load, *P_CS_*, and the stiffness in the crack stabilization stage, *K_CS_*, can be evaluated.

#### 3.4.2. Crack Stabilization Stage Behavior Evaluation by Regression Analysis

**Figure 15:**
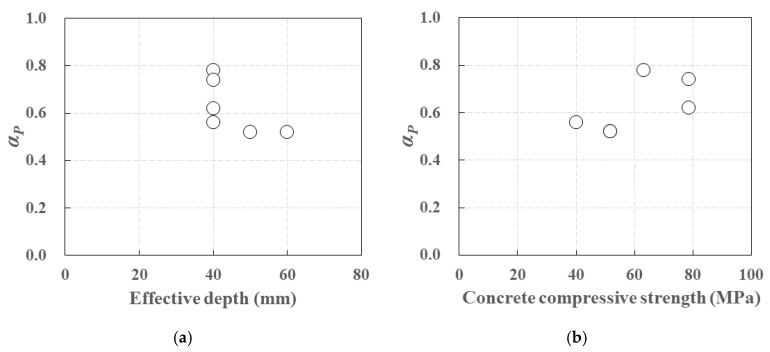
*α_P_* according to (**a**) effective depth, (**b**) concrete compressive strength.

The crack stabilization loads exhibited 0.52~0.78 of the maximum loads for the carbon-grid-reinforced specimens (Table 6). In addition, the ratio of the crack stabilization load to maximum load, *α_P_*, (Figure 15a) tended to decrease with the effective depth and increase with the concrete compressive strength (Figure 15b).

**Figure 16:**
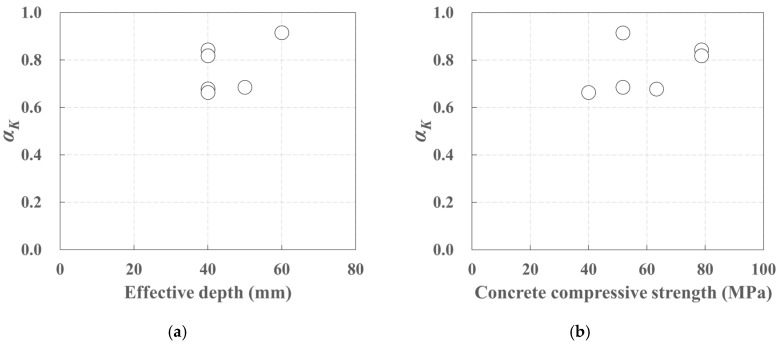
*α_K_* according to (**a**) effective depth, (**b**) concrete compressive strength.

The stiffness decreased to 0.004~0.390 of the initial stiffness due to crack initiation, and in the crack stabilization stage the stiffness further decreased to 0.663~0.915 of the stiffness in the crack formation stage (Table 7). The ratio of the stiffness in the crack formation stage to the stiffness in the crack stabilization stage, *α_K_*, (Figure 16a) also tended to increase with both the effective depth and the concrete compressive strength (Figure 16b).

Accordingly, *α_P_* was considered to be inversely proportional to the effective depth and proportional to the compressive strength of the concrete, and *α_K_* was considered to be proportional to both the effective depth and the concrete compressive strength. Based on the relationships between *α_P_* and the effective depth and the concrete compressive strength, as well as between *α_K_* and the effective depth and the concrete compressive strength, multiple linear regression models were developed with a 95% confidence interval using Microsoft Excel Spreadsheets^®^ 2016 [48]. The statistical models took *α_P_* and *α_K_* as dependent variables, with the effective depth and the concrete compressive strength as independent variables. The equations of the statistical models are stated in Equations (5) and (6) as follows:

(5) $$\alpha_{P}=-0.006d+0.003f_{cu}+0.725$$

(6) $$\alpha_{K}=0.010d+0.005f_{cu}$$

where *d* is the effective depth (mm) and *f_cu_* is the concrete compressive strength (MPa).

The values of the correlation coefficient and coefficient of determination in Equation (5) were 0.768 and 0.591, respectively. The values of the correlation coefficient and coefficient of determination in Equation (6) were 0.998 and 0.996, respectively.

**Figure 17:**
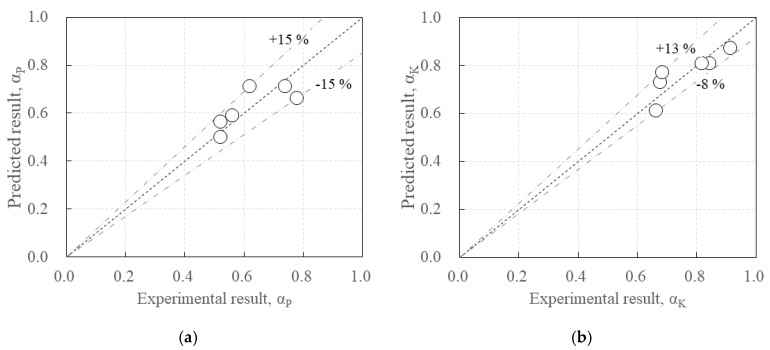
Comparison of predicted results with experimental results: (**a**) *α_P_*, (**b**) *α_K_*.

Table 8 summarizes the predicted values of *α_P_* and *α_K_*, and Figure 17 demonstrates a comparison of the predicted values of *α_P_* and *α_K_* with the experimental data.

*α_P_* exhibited a relatively weak linear relationship with effective depth and concrete compressive strength compared with *α_K_*, but Equation (5) was able to predict the experimental data within a ±15% range (Figure 17a). On the other hand, *α_K_* exhibited a strong linear relationship with effective depth and concrete compressive strength. Equation (6) was also able to predict the experimental data in a range between +13 and −8% (Figure 17b).

Table 8 also summarizes the calculated values of *P_CS_* and *K_CS_* based on the predicted values of *α_P_* and *α_K_*.

The ratios of the experimental to calculated crack stabilization loads ranged from 0.79 to 1.33. For specimen 30_KC_d40, which had the highest concrete compressive strength, the calculated crack stabilization load was 1.33 times higher than the experimental crack stabilization load. For specimen 40_KC_d60, which had the largest effective depth, the calculated crack stabilization load was 0.79 times lower than the experimental crack stabilization load. However, the calculated crack stabilization loads were generally similar to the experimental crack stabilization loads. On the other hand, the ratios of the experimental to calculated stiffnesses in the crack stabilization stage ranged from 0.85 to 1.53. The calculated stiffnesses in the crack stabilization stage gave similar results to the experimental stiffness in the crack stabilization stage for the under-reinforced specimens. In contrast, for the over-reinforced specimens, the calculated stiffnesses in the crack stabilization stage were higher than the experimental stiffnesses in the crack stabilization stage.

**Figure 18:**
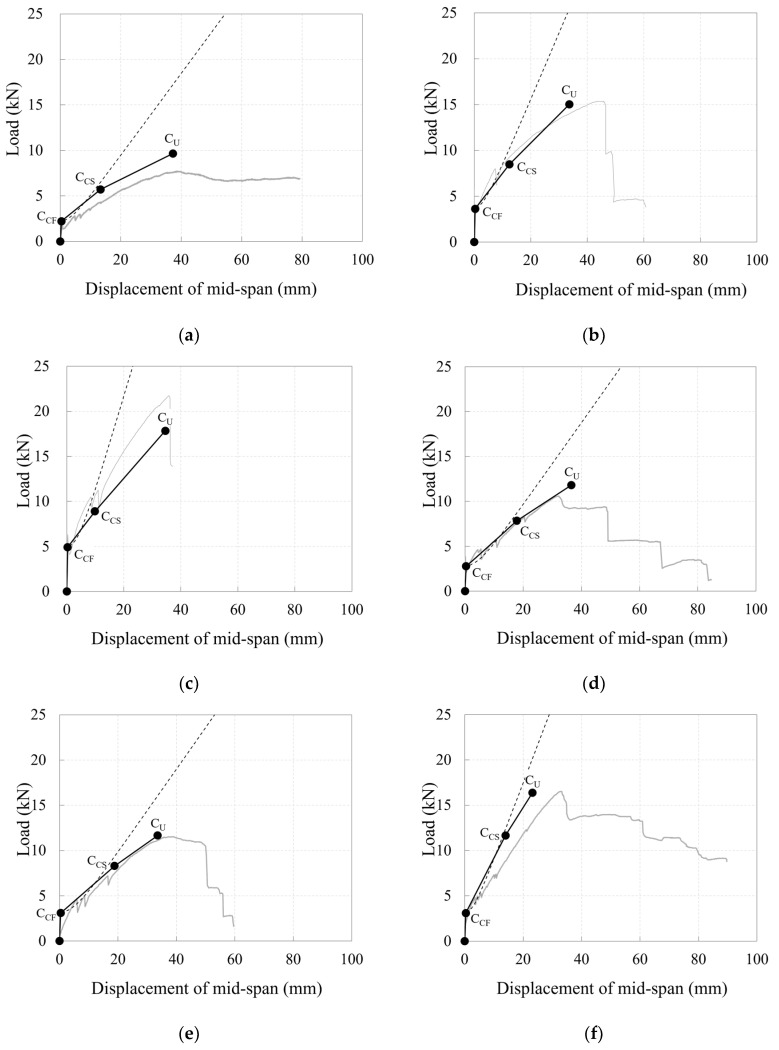
Comparison of the flexural behaviors of specimens: (**a**) 30_KC_d40; (**b**) 40_KC_d50; (**c**) 40_KC_d60; (**d**) 50_KC_d40; (**e**) 60_KC_d40; (**f**) 60_KC2_d40.

As demonstrated in Figure 18, the trilinear load–deflection curve, obtained based on the calculated characteristic values of loads, deflections, and stiffnesses for each stage according to Section 3.4.1 and Section 3.4.2, provided a relatively appropriate and accurate assessment of the flexural behavior in the carbon-grid-reinforced specimens. In Figure 18, the dashed curve refers to the load–mid-span relationships obtained using Equations (3) and (4), taking into accounting the decrease in stiffness reduction due to the weak bond between the carbon-grid reinforcements and the concrete [35], and has been shown to be applicable to the assessment of the crack formation stage behavior in the specimens. The solid curve also refers the load–mid-span relationships from the experiment (Figure 10).

The trilinear load–deflection curves of specimens 30_KC_d40 and 60_KC2_d40, which were over-reinforced with carbon grids and failed with concrete crushing, overestimated the experimental load–mid-span relationships. This is likely because the calculated values of *K_CF_* and *K_CS_* became higher than those of the experimental results. As a result, the trilinear load–deflection curve was shown to have a larger load at the same deflection. In contrast, for specimens 40_KC_d60, 50_KC_d40, and 60_KC_d40, which were under-reinforced with carbon grids, and specimen 40_KC_d60, which was over-reinforced with carbon grids but failed with the rupture of the CFRP strands, the trilinear load–deflection curves underestimated the experimental load–mid-span relationships which resulted in a smaller load at the same deflection.

### 3.5. Comparison of Flexural Behaviors with RC

**Figure 19:**
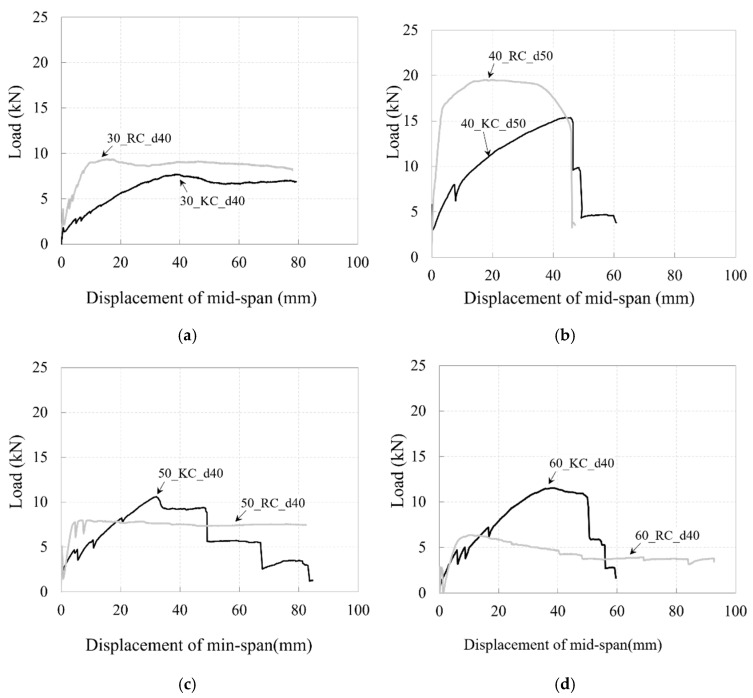
Flexural behavior of specimens reinforced with carbon grids and rebar: (**a**) C30; (**b**) C40; (**c**) C50; (**d**) C60.

Figure 19 depicts a comparison of the load–mid-deflection relationships for the carbon-grid-reinforced specimens with those of the RC specimens.

Compared with the RC specimens, the flexural stiffness of the carbon-grid-reinforced specimens decreased significantly after cracking. This is likely due to the weaker bond between the carbon grid and concrete, even though the surface embossing treatment of the carbon grid appeared to improve the bond performance to a certain extent based on the surface damage pattern. The low bond strength compared to that of the rebar reduces the tension-stiffening effect in concrete [5,47,49], and the thin plate-shaped cross-section of the CFRP strands in the carbon grid also contributes to this reduction in flexural stiffness. The differences between the flexural stiffnesses of the carbon-grid-reinforced and RC specimens tended to decrease as the effective depth or concrete compressive strength increased.

Additionally, among the specimens with relatively high-compressive-strength concrete (C50 and C60), the maximum loads of RC specimens 50_RC_d40 and 60_RC_d40 were 80% and 64% of the designed flexural strength, respectively (Table 4), indicating poor performance. In contrast, the carbon-grid-reinforced specimens, 50_KC_d40 and 60_KC_d40, reached 99% and 101% of their designed flexural strengths, respectively, at the maximum load. As a result, the maximum loads of carbon-grid-reinforced specimens 50_KC_d40 and 60_KC_d40 were 1.33 and 1.81 times higher, respectively, compared to those of RC specimens 50_RC_d40 and 60_RC_d40. However, unlike the RC specimens, the carbon-grid-reinforced specimens exhibited brittle failure due to the rupture of the longitudinal CFRP strands after reaching the maximum load.

For the specimens with relatively low-compressive-strength concrete (C40), although carbon-grid-reinforced specimen 40_KC_d50 was designed with 1.13 times the flexural strength of RC specimen 40_RC_d50, its maximum load was only 0.79 times that of the RC specimen. Similarly, although carbon-grid-reinforced specimen 40_KC_d60 was designed with 1.37 times the flexural strength of RC specimen 40_RC_d50, its maximum load was only 1.11 times that of the RC specimen, suggesting that the actual flexural performance of 40_KC_d60 approached that of 40_RC_d50. This is because carbon-grid-reinforced specimens 40_KC_d50 and 40_KC_d60 achieved 1.02 and 1.22 times their design flexural strengths, respectively, while RC specimen 40_RC_d50 reached 1.47 times its designed flexural strength. Additionally, RC specimen 40_RC_d50 exhibited a brittle failure behavior similar to that of the carbon grid-reinforced specimens as it also failed because of rebar rupture after reaching the maximum load.

## 4. Conclusions

In this study, the performance of carbon grid KC, whose surface was embossed to increase bonding with concrete, was evaluated as a reinforcement for concrete via a three-point bending test. For this, one-way concrete slab specimens reinforced with carbon grid KC were fabricated. The variables included the effective depth, amount of carbon grid reinforcement, number of carbon grid layers, concrete compressive strength, and type of reinforcement. The results are concluded below.

1. The carbon-grid-reinforced specimens were considered to have improved bonding between the concrete and the CFRP strands due to the increased specific surface area from the surface embossing of the CFRP strands, based on the crack formation patterns, which resulted in no bond cracks and a distributed cracking pattern. Also, the specimens with higher concrete compressive strengths exhibited better bond performance.
2. For the specimens reinforced with one carbon grid layer, increasing the effective depth was more effective than increasing the concrete compressive strength for improving the flexural behavior, including the maximum load and stiffness, of the carbon-grid-reinforced members.
3. The ratios of the experimental to calculated flexural strengths for the carbon-grid-reinforced specimens ranged from 0.74 to 1.22 and were also 1.22–2.22 times higher than the strength reduction factor specified in the design standards. As the amounts of carbon-grid reinforcement increased, the ratio decreased for the under-reinforced specimens but increased for the over-reinforced specimens.
4. Based on the experimental results, this study proposed a trilinear load–deflection curve to assess the flexural behavior of carbon-grid-reinforced concrete members. The trilinear load–deflection curve with the calculated characteristic values closely simulated the flexural behavior observed in the experiment.
5. Although the RC specimens exhibited lower performance than the design expectations, the carbon-grid-reinforced specimens met the design performance requirements, showing similar post-crack stiffness and higher maximum loads than the RC specimens. Therefore, for carbon-grid-reinforced members with relatively small effective depths and high concrete compressive strengths, as presented in this study, a favorable performance comparable to that of RC members can be expected.

These results indicate that carbon grid KC exhibits the necessary performance to act as a reinforcement for concrete, and a flexural design following the standards for GFRP reinforcements appears to be feasible. However, the results are based on limited experimental data, and further experimental and analytical studies are required, along with a comprehensive analysis of the existing data, for the design of carbon-grid-reinforced flexural members in the future.

## Figures and Tables

**Table 1:** Characteristics and tensile properties of reinforcements.

Name	Strand			Grid Geometry (Longitudinal (G__VS_) × Transverse (G__TS_) Spacing, mm)	Tensile Strength (MPa)	Tensile Modulus of Elasticity (MPa)
	Width (mm)	Thickness (mm)	Area (mm^2^)			
KC	10	1	10	100 × 100	2327	209,069
d10	-	-	71.3	-	553 (452)	177,000

() yield strength.

**Table 2:** Mix designs and compressive strengths of concrete.

Name	Cement (kg/m^3^)	Water (kg/m^3^)	W/C (%)	Fine Aggregate (kg/m^3^)	Coarse Aggregate (kg/m^3^)	Compressive Strength (MPa)
C40	482	182	38	796	985	51.7
C50	570	172	30	774	959	63.3
C60	658	165	25	748	928	78.7

**Table 3:** Specifications of one-way slab specimens.

Specimen	Width (mm)	Depth/ Effective Depth (mm)	Length (mm)	Reinforcement	Reinforcement Ratio (%)	Concrete
40_KC_d50	300	60/50	1000	KC	0.20	C40
40_KC_d60		70/60			0.17	
50_KC_d40		50/40			0.25	C50
60_KC_d40		50/40			0.25	C60
60_KC2_d40		50/40			0.50	
40_RC_d50		80/50		d10@200 (longitudinal direction) × d10@200 (transverse direction)	0.70	C40
50_RC_d40		70/40			1.19	C50
60_RC_d40		70/40			1.19	C60

**Table 4:** Crack and flexural strength calculation results for specimens.

Specimen	$\rho /\rho_{b}$	Crack Strength		Flexural Strength		Failure Mode
		Moment (kNm)	Load (kN)	Moment (kNm)	Load (kN)	
40_KC_d50	1.35	815	3.62	3384	15.04	Concrete crushing
40_KC_d60	0.69	1110	4.93	4012	17.83	CFRP strand rupture
50_KC_d40	0.96	627	2.78	2654	11.80	CFRP strand rupture
60_KC_d40	0.94	699	3.10	2623	11.66	CFRP strand rupture
60_KC2_d40	1.87	699	3.10	3687	16.39	Concrete crushing
40_RC_d50	0.44	1450	6.44	2994	13.30	Steel yield
50_RC_d40	0.76	1228	5.46	2224	9.89	Steel yield
60_RC_d40	0.74	1369	6.09	2232	9.92	Steel yield

*ρ: reinforcement ratio, ρ_b_: reinforcement ratio producing balanced strain conditions.*

**Table 5:** Crack formation in specimens.

Specimen	Number of Cracks
40_KC_d50	2
40_KC_d60	3
50_KC_d40	5
60_KC_d40	5
60_KC2_d40	6
40_RC_d50	3
50_RC_d40	3
60_RC_d40	2

**Table 6:** Load and deflection of mid-span results.

Specimen	Crack Formation Point (C_CF_)		Crack Stabilization Point (C_CS_)		Maximum Load Point (C_U_)		Load at C_CF_/Load at C_U_	Load at C_CS_/Load at C_U_
	Load (kN)	Deflection of Mid-Span (mm)	Load (kN)	Deflection of Mid-Span (mm)	Load (kN)	Deflection of Mid-Span (mm)		
30_KC_d40 [35]	1.80	0.67	4.31	13.19	7.70	38.69	0.23	0.56
40_KC_d50	5.80	−0.01	8.00	7.49	15.37	44.01	0.38	0.52
40_KC_d60	6.32	0.37	11.25	11.01	21.75	35.77	0.29	0.52
50_KC_d40	2.16	0.03	8.23	20.43	10.59	32.09	0.20	0.78
60_KC_d40	4.70	5.96	7.20	16.54	11.53	38.33	0.41	0.62
60_KC2_d40	3.47	−0.01	12.28	21.10	16.52	32.92	0.21	0.74
30_RC_d40 [35]	3.87	3.87	5.94	4.60	9.38	15.49	0.41	0.63
40_RC_d50	-	-	12.01	2.09	19.52	18.05	-	0.62
50_RC_d40	5.07	0.29	6.19	3.00	7.96	9.40	0.64	0.78
60_RC_d40	2.89	0.18	4.14	4.28	6.37	10.46	0.45	0.65

For the RC specimens, the crack stabilization state refers to the point at which steel yielding occurred.

**Table 7:** Stiffness results.

Specimen	Stiffness (N/mm)			Stage 1/Stage 3	Stage 2/Stage 3
	Before Crack Formation (Stage 1)	Crack Formation (Stage 2)	Crack Stabilization (Stage 3)		
30_KC_d40	2687	200	133	0.075	0.663
40_KC_d50	-	294	202	-	0.685
40_KC_d60	17,081	463	424	0.027	0.915
50_KC_d40	71,833	298	202	0.004	0.678
60_KC_d40	789	236	199	0.299	0.843
60_KC2_d40	-	418	359	0.390	0.818
30_RC_d40	8019	-	-	-	-
40_RC_d50	-	-	-	-	-
50_RC_d40	17,789	-	-	-	-
60_RC_d40	16,486	-	-	-	-

**Table 8:** Predicted results by multiple linear regression analysis.

Specimen	$\alpha_{P}$	$\alpha_{K}$	$P_{CS}$ (kN)	$K_{FS}$ (N/mm)	$K_{CS}$ (N/mm)	Calculation Results/Experimental Results		
						$P_{CS}$	$K_{FS}$	$K_{CS}$
30_KC_d40	0.59	0.61	5.71	270	165	1.33	1.35	1.24
40_KC_d50	0.56	0.77	8.47	401	309	1.06	1.36	1.53
40_KC_d60	0.50	0.87	8.89	414	361	0.79	0.89	0.85
50_KC_d40	0.66	0.73	7.83	290	212	0.95	0.96	1.05
60_KC_d40	0.71	0.81	8.30	282	282	1.15	1.20	1.15
60_KC2_d40	0.71	0.81	11.67	632	632	0.95	1.44	1.42

## Data Availability

The original contributions presented in the study are included in the article, further inquiries can be directed to the corresponding author.
